# Development of a New Scale to Assess Students’ Autodetermination At School (AAS)

**DOI:** 10.3390/ejihpe14010012

**Published:** 2024-01-06

**Authors:** Christine Sanchez, Bertrand Porro, Nathalie Blanc

**Affiliations:** 1 Department of Psychology, University Paul Valéry Montpellier 3, EPSYLON UR 4556, 34000 Montpellier, France; nathalie.blanc@univ-montp3.fr; 2 Université d’Angers, Université de Rennes, Inserm, EHESP, IRSET (Institut de Recherche en Santé, Environnement et Travail) UMR_S 1085, IRSET-ESTER, SFR ICAT, F-49000 Angers, France; bertrand.porro@univ-angers.fr; 3 Department of Human and Social Sciences, Institut de Cancérologie de l’Ouest (ICO), 49055 Angers, France

**Keywords:** self-determination theory, commitment, school, scale validation, AAS

## Abstract

The Autodetermination At School (AAS) hetero-evaluative scale was created and validated in a French teacher population with the aim of quantifying, in an ecological way, the commitment at school of elementary students. After establishing the scale’s face validity, AAS was tested with an exploratory factor analysis, a confirmatory factor analysis, a convergent validity analysis, a test–retest analysis and an inter-individual analysis. The EFA highlighted three distinct factors and the CFA validated the reliability of a three-factor model for AAS with relevant fits and indices. The first dimension concerns teacher perception of academic commitment, reflecting both child performance and autonomous motivation. The second and third ones reflect teacher perception of the child’s social commitment, to their peers as well as to their teacher. Consequently, AAS is a useful, reliable and robust psychometric instrument that emphases how intrinsic motivation and performance are closely linked. It also considers the importance of social child commitment at school. The inter-individual analysis revealed trends of grade, gender and school environment effects.

## 1. Introduction

The classroom represents the first real social context [1] where children develop outside the family environment. Therefore, it is of major importance for children to feel good at school to engage in efforts in learning and to fully develop their potential. In this sense, child commitment is one of the key components to consider in helping them to experience learning in a positive way. If there is a lack of commitment in school, solutions should be explored to address this issue. In other words, it is crucial to understand what various metrics are available to measure students’ commitment. One challenge that still needs attention is to bridge the significant gap between the importance of commitment in the school environment and the scarcity of existing easy-to-use hetero-evaluative scales assessing students’ commitment in its various components. This challenge is the core of this study, which consists of the creation of a scale. Please note here that the terms autodetermination and self-determination will also be used in this article to talk about commitment, these terms referring to a form of psychological freedom to invest that allows commitment.

### 1.1. The Importance of Students’ Commitment

Various reasons and theoretical models justify questioning the notion of engagement in general, and the notion of student learner engagement in particular. Considering intelligence and the development of potentialities leads in fact to an interest in commitment. According to the three-ring theory [2], commitment is crucial as one of the three components along with aptitudes and creativity that contributes dynamically to the optimal development of an individual’s intelligence [2,3,4]. In this sense, the self-determination Theory (SDT) of Ryan and Deci [5,6] appears fundamental in understanding the multidimensional concept of commitment through the prism of psychology. The SDT, which proposes a conceptual framework that investigates the various sources of motivation, refers to the understanding of autodetermination that precedes commitment. This empirical organic macro-theory [5,6] is based on a broader concept of motivation, individual development and well-being [7]. The SDT suggests that individuals usually experience a thirst for learning to assimilate knowledge and values in order to better grasp the world around [8]. “All men by nature desire to know”. Referring to Aristote, Deci [9] conceptualized intrinsic motivation starting from an obvious observation: the child is spontaneously insatiable and oriented towards knowledge. In such a model, motivation and the satisfaction of basic psychological needs (i.e., the need to feel competent, autonomous and connected to others) condition well-being (Chartier, 2018). Most importantly, the SDT defines commitment as corresponding to all active human behavior and mechanisms that underlie personality development and construction [6]. In this regard, supporting or eliciting the learner’s psychological enthusiasm or commitment seems the best way to achieve qualitative and sustainable learning [8,10]. In fact, this support for commitment (and therefore for self-determination, which seems inseparable from commitment) appears to be a fundamental concept in the psychological literature on learning. Commitment is indeed intimately linked to well-being at school [10], to achievement [11,12], and to the prevention of dropout rates [11,12,13]. Also, if the SDT has applicability perspectives in a plurality of fields [6,7], “SDT is of much importance in the domain of education, in which students’ natural tendencies to learn represent perhaps the greatest resource educators can tap” [8] (p. 134), according to Niemiec and Ryan [8].

### 1.2. Engaging Students: The Question of Motivation

Commitment encompasses a set of components that influence a child’s development in the school environment. Overall, SDT states that self-determination at school is a multidimensional datum with different interrelated components interacting in a dynamic and reciprocal way. Among these components, motivation (i.e., dynamic process underlying the initiation, direction, intensity, and persistence of behavior), is the entry point for task commitment and even more broadly, for academic and professional success [14]. The SDT posits that it is indeed important to nurture individual motivational tendencies, which initially lead the child, spontaneously, to learn for growth [15]. However, within the SDT, the conception of motivation has evolved significantly. Traditionally, three types of motivations were considered [15]: intrinsic motivation (i.e., natural, without pressure or external incentives), extrinsic motivation (i.e., responding to more or less internalized external demands), and amotivation (i.e., loss or total lack of motivation). Nowadays, without considering these conceptions as obsolete, there is a greater distinction made between autonomous motivation (which encompasses both intrinsic motivation and other forms of internalized extrinsic motivations, provided they are uncontrolled and aligned with personal values, needs, or choices), controlled motivation (used to describe an individual’s non-internalized extrinsic motivation), and the concept of amotivation [16]. Also, in order to foster the emergence of engaging autonomous motivation, there would be a need to respect the basic psychological needs of competence, relatedness and autonomy. The theory of basic psychological needs (BPNT) is one of the central mini-theories of the SDT [17,18,19]. The need for competence refers to the intrinsic necessity to perceive oneself as effective in one’s environment, meaning being capable of mastering meaningful tasks and developing personal skills. The need for autonomy resides in the support provided for the individual’s intrinsic quest for autonomy in action, where one must feel capable of making meaningful choices that position him/her as the originator of their own actions. The satisfaction of the need for social connection involves the natural pursuit of meaningful interpersonal connections, social support, and emotional bonds, considered one of the conditions for psychological well-being [16,17,18,19]. In this respect, the quest to satisfy basic psychological needs can be a real lever for action for educational staff, with a view to stimulating motivation and self-determination in students. When teachers want to satisfy students’ basic psychological needs, they have better outcomes in terms of learning autonomy, academic performance and even considering well-being [8]. In summary, the SDT has enabled the accumulation of knowledge that encourages the development of educational and teaching styles that support the consideration of motivation and psychological needs in the school context [20,21]. In this sense, the SDT makes a clear distinction between the bright path and the dark path of teaching [19,20,21,22]. Bright teaching supports and satisfies needs, thereby promoting engaging autonomous motivation. Dark teaching, on the other hand, hinders needs, frustrates them, and at best generates controlled motivation, if not a form of clearly disengaging amotivation for the learner.

### 1.3. Measuring Engagement: An Overview of Existing Tools

Up to now, there are no easy-to-use hetero-evaluative scales to assess school commitment (i.e., no quick-to-fill tools, that are therefore non-invasive in school). Some interesting measurement systems are already available with which to probe some components of school commitment. Ryan and Deci’s General Causality Oriented Scale appears as the most comprehensive one, dealing with motivation based on the adoption of autonomous, controlling or impersonal behaviors [23,24]. With the Academic Motivation Scale (AMS), available in several versions, motivation is measured in all its forms, in various contexts [24,25,26]. Such scales assess motivation [23,24,25,26] in a more exhaustive and precise way than they measure child commitment in its entirety to help them with schooling. The child’s engagement or re-engagement after a problem situation is also at stake in a system that should not ignore motivational resilience [27]. This is not a measure of commitment per se, but one of the predictors of engagement (i.e., including motivational resilience). Recently, the BPNSS (Basic Psychological Need Satisfaction Scales), the aim of which is to evaluate the fundamental psychological needs of students, draws links with autodetermination, but still does not measure it directly and entirely [12]. The School Engagement Measure (SEM) [28] scale, on the other hand, is an interesting tool for assessing a child’s school engagement. However, it is self-reported, validated for adolescents, and does not take into account, like other self-reported scales such as the EDES (Scale dimensions of school Engagement; [29] or the French FAS (Feelings About School), the child’s sense of academic competence by considering different academic subjects independently [30].

As a consequence, with the creation of the Autodetermination At School scale, our goal was to develop a hetero-evaluative scale for assessing a child’s school engagement that could be adapted for evaluation by teachers, for children aged 6 to 12 years, taking into account both the importance of the student’s autonomous motivation and the satisfaction of their needs to feel competent and connected with their teacher and peers. The AAS scale was designed by taking into account both the SDT applied to educational practice [8] and existing scales, in particular those dealing with hierarchical motivation (i.e., with an understanding of motivation that considers several types of motivation as in Section 1.2) [26,31].

## 2. Method: Design of the AAS Scale and Procedure

### 2.1. Modalities for AAS Creation

#### 2.1.1. A Synthetic and Hetero-Evaluative Scale

The AAS scale is an ecological compact scale to assess a child’s Autodetermination At School. It is hetero-evaluative because teachers and observers can provide robust reports on student engagement in the classroom [13]. This hetero-evaluative tool also allows for multimodal measurements, with functional and more subjective self-reporting scales, such as feelings at school based on self-system theory, for example [18,30]. Requiring the teacher’s participation for 5 min, the AAS scale is extremely quickly to-use to not overload teachers, psychologists, or other child professionals, avoiding a too-demanding task in terms of time investment (i.e., feasibility). Based on the theories mentioned above, the AAS scale was built to encourage intra-professional collaboration between teachers and psychologists. This scale is composed of 11 items before validation, corresponding to the objective to validate an easy-to-use tool to reveal a synthetic point of view [32].

#### 2.1.2. A Scale to Measure Autodetermination At School for 6- to 12-Year-Old Children

In France, elementary school spans five years, covering a large part of the period of education from 6 to 12 years of age. It seemed relevant to create a scale that could measure the student’s commitment throughout their elementary school education, and to assess it over a large period, which is known to be crucial to predict their education trajectory. This scale could serve as an exclusive reference for engagement in elementary school, particularly for National Education Psychologists EDA (i.e., specialists in supporting student difficulties from entry into preschool at 3 years old to the end of elementary school). Overall, it aims to cover an interesting period (from 6 to 12 years) from the perspective of research on engagement in learning, which is a crucial period during which the transition from primary to secondary learning occurs (Tricot, 2016). Learning experiences encountered in the preschool years up to six years old require intrinsic motivation that is innate because they are enjoyable and adaptive. Starting from six years old, a child’s entry into elementary school marks a progression towards conscious secondary learning. During this phase, it becomes more challenging for the child to persevere without support aimed at transforming controlled extrinsic motivation (e.g., “I have to bother decoding to avoid bad grades”) into autonomous extrinsic motivation (e.g., “I need to persist in decoding because it will soon allow me to discover many wonderful worlds on my own in the library books”).

Elementary school is therefore a key stage in the development of autonomous motivation, enabling the child to succeed in their learning. In this regard, we choose to create and validate a scale for measuring self-determination in school for this age group.

#### 2.1.3. A Multidimensional Scale

Before thinking about how to assess school commitment, it was first necessary to consider engagement as multi-sided [33]. The scale was constructed by considering three factors based on basic psychological need satisfaction as outlined in the SDT applied to education: competence, autonomy and sociability [6]. The first factor includes the notion of autonomy through the consideration of a child’s autonomous motivation in a learning situation. The second one transcribes competences through the evaluation of academic performances. The third focuses on the child’s social abilities. For each of these dimensions of the SDT, there are at least three items that are proposed to elaborate the structure of our scale (Table 1).

### 2.2. The AAS Scale’s Composition

#### 2.2.1. Student’s Autonomous Motivation (3, 5, 7, 10)

The first dimension could bring together four items: item 3 (interest for schoolwork), item 5 (pleasure to learn), item 7 (autonomy) and item 10 (perseverance). Those items refer to the learner’s autonomous motivation by exploring notions like interest, pleasure, autonomy and tenacity.

Autonomous motivation (including intrinsic motivation and internalized extrinsic motivations) differs from other forms of motivation like extrinsic controlled motivation and amotivation. Intrinsic motivation is one of the motivational forms found in autonomous motivation because it values experience for itself, for the pleasure of learning. Intrinsic motivation can be understood as conditional on the ideal appearance of a learning child’s volition. However, the internalized (and therefore uncontrolled) forms of extrinsic motivation found in autonomous motivation also play a part in the learner’s volition, since they enable the learner to see a task as meaningful, or even as being in line with his values. Some authors have used the SDT principle to show how intrinsic motivation is one of the fundamental pillars for qualitative and beneficial learning [34]. A longitudinal study has actually reaffirmed the power of reciprocal links [6]. Indeed, they noticed how much autonomy support and the satisfaction of the need for independence, autonomy and intrinsic motivation, intrinsic motivation and commitment, and finally involvement and academic success are all strongly linked. However, the idea that all children have a natural propensity to turn to knowledge and learning [8,9] does not mean that school can really capitalize on this reality [6]. In the end, autonomous motivation conditions the child’s spontaneous involvement in a task, and evaluating children’s autonomous motivation in the school environment is important because it predicts well-being and academic success, and is even linked to the development of creativity [35].

Engagement in learning underlies chances of successful instruction and notably reflects the learner’s intrinsic motivation [10]. Item 3 (interest) and item 5 (pleasure to learn), aimed at measuring essential elements to intrinsic motivation, are also found in the AMS scale [24]. Pleasure and interest refer to the crucial idea of fulfillment. Interest is described as correlated with intrinsic motivation [36] and refers more specifically to intrinsic motivation for knowledge (e.g., IM-to-know), [26]. As for the notion of pleasure, on one hand it refers to well-being, known to be highly correlated with intrinsic motivation [37]. On the other hand, it is about succeeding to preserve a child’s innate propensity to learn from an early age, based on learning experience stimulation [6,8,9]. In other words, it is about children’s IM to experience stimulation [26]. Moreover, pleasure, which is introduced by the formula “For the pleasure (…)”, is associated with the intrinsic motivation for achievement, knowledge and experiential stimulation in the AMS in five items (questions 6, 11, 13, 16 and 18 in [31]). Item 7 (Autonomy), which refers directly to self-determination and therefore to intrinsic motivation through autonomous motivation, is important because SDT authors have demonstrated the impact of autonomy support on student’s intrinsic motivation [24,38]). Autonomy is one of the three fundamental psychological needs for individual development [6]. Ryan and Deci [6] recently reaffirmed how much autonomy is associated with a higher degree of commitment and performance no matter the education level. Item 10 (perseverance) shows a child’s possible discouragement or ability to sustain effort demonstrating, tenacity associated with autonomous motivation [14]. This pugnacity found in the AMS (question 20 in [31]) is taken as an evidence of motivation intensity, which animates a child learner and allows him to overcome difficulties.

#### 2.2.2. Student’s Academic Performance or Abilities (1, 6, 9)

The second factor for which item 1 (mathematics), item 6 (graphical ability) and item 9 (literacy) were designed to measure the child’s involvement through teacher perception of performance in different academic areas is a way to explore competence, another fundamental psychological need for favorable individual development [6,8,24]. Because academic results are also very reflective of learning success, our scale simply measures child performance in mathematics, art, graphical ability and literature. It is a means of specifically assessing a child’s intra-individual progression, either to help him or her, to target and value if he or she is in difficulty, or as part of a longitudinal study.

#### 2.2.3. Student’s Adjustment or Social Commitment (2, 4, 8, 11)

The SDT concerns both intrinsic individual mechanisms and its expression in a social context [6,24]. The SDT also deals with an individual’s abilities to interact and forge qualitative and lasting social links; it is the third fundamental schoolchild psychological need. This is why the question of a child’s social adjustment is crucial in our measurement scale. In the theoretical framework of motivation dynamics [24], interpersonal relationships appear to be a very strong determinant of motivational context. The same observation can be made in this hierarchical motivation organization system: the motivational context conditions an individual’s effective overall motivation. The academic context substantially impacts a child’s involvement. Extrinsic forms of motivation like support for autonomy and giving meaning to the task may then sustain the child’s involvement. Some extrinsic forms of motivation are more likely to help a child internalize the need to go through learning, which cannot always be very attractive in itself [8]. Ask a very young child to repeatedly draw vertical lines in his notebook; he may find it boring and give up the task. If you notify him that when they know how to do this, they will be able to write the first letter of their first name, they might be more likely to master drawing of a vertical line even if it takes repeated learning. At school, the child’s relationship with his teacher and peers and his abilities to connect with others is naturally an essential condition for a motivating context. The more qualitative the links are, the more a child will internalize academic learning procedures. That is why the third factor conceptualized aims to evaluate the child’s degree of social adjustment. Item 2 (adjustment to teacher) and item 11 (link with student) assess the relationship with teacher. Item 4 (integrative skills) and item 8 (adjustment to peers) measure the relationship with peers. The child’s relationship with the teacher has a significant impact on the effective motivation, autonomy and therefore engagement in the school environment, both academically and socially. Supported, a child who learns independently and confidently, without control, will be much more motivated according to the SDT [8].

#### 2.2.4. Design of Items

The items have been designed for maximum clarity (in terms of syntax, vocabulary, and the absence of ambiguity or socially desirable effects) to facilitate understanding by teachers and to avoid biases. The items are intended to be effective and to comprise on average 11 words. For the sake of consistency and idiomatic simplicity, we used the same initial formulation (i.e., ‘How would you rate his/her…’) for nine of the designed items (1, 2, 3, 4, 5, 6, 8, 9, and 11). These nine items indeed assess a capacity, a trait, or a behavioral adjustment on a scale ranging from nonexistent or below expected acquisition (for zero) to total or well above expected acquisition (for a score of 10). Items 7 and 10 were formulated differently because they pertain to the evaluation of the frequency or recurrence of observing autonomy behavior (item 7) and a perseverant attitude (item 10) in the child facing the task, rather than a comprehensive assessment of autonomy and perseverance. Item 5, which relates to the notion of learning (from ‘a lot of displeasure’ for zero to ‘a lot of fun’ for 10), is also an evaluation of the child in a learning situation and is not a comprehensive assessment of the pleasure experienced by the child at school. Given its subjective nature, this evaluation explains why we acknowledged the subjectivity of the response in the initial wording of the item (i.e., ‘How do you think he/she feels...’).

#### 2.2.5. Mixing of Items

There are several items for involvement in each factor. For example, there are 4 items (3, 5, 7, and 10) on motivation. We mixed the above items to avoid a possible order effect. This is a way to increase the chances of a teacher focusing on one item at a time and not on a previously given answer.

#### 2.2.6. Likert Scale and Track Bar

To maximize scale sensibility and increase chances of observing an evolution, even a minimal one, a track bar is used. It is a continuous line [39] from 0 to 10 to maintain a grading system familiar to French teachers. Initially, the cursor was positioned at the track bar’s center. For each item, participants simply had to move the cursor between 0 and 10 to evaluate their student. The easy-to-use track bar allowed for more accurate results. It should also limit the risk of having a learning effect and an order effect when the questionnaire is filled out several times by the same teacher. The questionnaire was created and completed online.

### 2.3. Procedure for AAS Scale Validation

We report how we determined our sample size, all data exclusions, all manipulations, and all measures in the study.

#### 2.3.1. Study Design

The validation of the scale required a cross-sectional collection of data. The AAS scale was designed using a secure and private computer tool that meets ethical standards for data retention. The interface was designed for data collection through questionnaires allowing several phases of participation to be included with a pre-selection phase based on inclusion criteria for respondents. It is easy to provide and broadcast the questionnaire link. Once the inclusion phase was over, the respondent discovered the 11 AAS scale items. The respondent had to answer all items by moving the track bar cursor, initially positioned in the middle. The questionnaire could not be validated until the respondent had answered all of the items.

#### 2.3.2. Participants

Our sample is divided in two, because the protocol for the qualitative validation of such a scale involves performing an EFA and CFA of it on two different populations. It is crucial to conduct a confirmatory factor analysis (CFA) on a separate sample from the one used for the exploratory factor analysis (EFA) because the aim of CFA is to confirm not the data themselves but a model that could work across different samples. In accordance with the recommendations [40], an EFA should be carried out with a minimum of 10 participants per questionnaire item evaluated (10:1) and a CFA should be carried out with a minimum of 20 participants per item evaluated (20:1). As the initial questionnaire comprised 11 items, a minimum sample of 110 students was required for the EFA and 220 students were required for the CFA. Precisely, N = 164 for the EFA sample and N = 361 for the CFA sample, which was also used for interindividual analysis.

#### 2.3.3. First Sample (for the EFA)

Each participant from the first sample was asked to fill out the AAS scale twice: once by thinking of a student “without any difficulty at school” and a second time, by thinking of a student “with difficulties at school”.

It was necessary to widely distribute the questionnaire so that it could be completed by as many school teachers as possible. After having solicited a direct network, a schoolteacher was recruited to social networks for more participation. Each participant had to be a schoolteacher in a French public school. This inclusion parameter was implemented by a simple and short question: “Are you a school teacher in a French public (elementary) school?”. A yes or no answer was expected. Indeed, there was no need to introduce other variables for this scale. It was intended to be effective for any French public elementary school teacher, regardless of gender, age or experience.

For the balance and quality of our scale’s validation, we wanted it to be both sensitive and valid. Therefore, we considered only the results of the 82 participants who completed the scale twice, once for a student without difficulty and a second time for a student with problems or particular needs. In summary, the AAS scale was therefore filled out 164 times in total by 82 participants.

#### 2.3.4. Second Sample (for the CFA and for Interindividual Analysis)

The second sample was composed of 52 teachers issued from the French public education system. They all exercise their profession in the first degree (i.e., which corresponds to first to fifth grade). We recruited them as part of another ongoing experiment dealing with learning optimization and fulfillment at school. Thus, 52 teachers answered the AAS scale for several of their students randomly selected in their class listing. Our AAS scale was completed by 52 respondents for N = 361 of their students.

We gathered additional data on most of these students, to conduct inter-individual analyses as a third step. These analyses examined the external validity (i.e., the robustness) of the scale and enabled us to understand the scale’s characteristics according to gender, level and school environment.

#### 2.3.5. Procedure for Each Sample

The procedure was slightly different for respondents in the first and second sample. All respondents had to carry out a study available by clicking on the link provided for this purpose and using a secure online platform specific to our laboratory, created to securely store the data collected. For the 82 participants from the first sample (who completed the questionnaire for two students each, for 164 questionnaires in total for the EFA), this study was conducted in three distinct steps: the admission phase, the completion of the questionnaire for a student without any difficulty, and finally the completion of the questionnaire a second time for a student with difficulties at school. For the other 52 participants from the second sample (for the CFA and interindividual analysis), they just had to fill out the questionnaire once for each of several students we randomly selected from their class listing (6 or 7 for each of the 52 teachers for 361 questionnaires completed in total). To answer each item, they had to move a cursor on a continuous line [39]. They could see how they positioned the cursor on the line but they could not see precisely the score out of 10 they assigned to students. Scoring for each item was performed automatically on the data collection platform, giving us a continuous score between 0 and 10. As the tool is designed to be used repeatedly for an individual follow-up or for research requiring testing and retesting, it seems relevant to enable the respondent to locate the child without seeing the exact score attributed. Many biases or learning effects can thus be avoided.

## 3. Statistical Methods

### 3.1. Hypothesis

The analyses were carried out with the aim of testing various hypotheses. First, we initially thought we could establish the face validity of our instrument with an audience already knowledgeable in psychology (i.e., N = 26 students in psychology). Secondly, the exploratory factor analysis aimed to allow us to validate the possibility of considering a three-dimensional construct for our scale. Thirdly, we hypothesized being able to validate the structure of our construct through a confirmatory factor analysis. Fourthly, our objectives were to demonstrate the convergent validity and test–retest reliability of the scale. Finally, the external validity of the scale had to be established through an ANOVA.

### 3.2. Face Validity

To establish the face validity of the idiomatic and semiotic aspects of our tool, we solicited the opinion of students (N = 10) who were masters of psychology and PhD students in psychology (N = 16), through an online questionnaire realized on the Qualtrics platform of the lab. The participants to this pre-test answered, after discovering the items of the scale, a set of yes/no questions concerning (i) the syntax and vocabulary; (ii) the clarity; (iii) the length of instructions and statements; (iv) possible grammatical errors; (v) discomfort in reading the items; (vi) the neutrality of the items; and (vii) social desirability bias.

### 3.3. Exploratory Factorial Analysis (EFA)

A set of preliminary analyses were first conducted considering the following: the normality of each item (Shapiro–Wilk test), multivariate normality (Doornik Hansen test), the correlation matrix, the Kaiser–Meyer–Olkin index (KMO—needed to be close to 1), Bartlett’s test (the *p*-value needed to be less than 0.05), and parallel analysis (using the SPSS package parallel.sps). Then, we conducted an exploratory factor analysis. The principal axis method followed by Promax rotation with Kaizer normalization for oblique factors was used due to the correlated nature of the dimensions. After rotation, items were retained if their unique variance was <0.80, their factor loading was >0.40, or cross-loadings were <0.30 on a second factor.

### 3.4. Confirmatory Factorial Analysis (CFA)

CFA was performed using structural equation modeling (SEM) from the variance–covariance matrix. From the results of the EFA, several models were evaluated. The multivariate normality assumption was first checked using the Doornik–Hansen test. For each model, a robust estimation method (i.e., the Satorra–Bentler method; [41]) was performed. The estimated models were compared using the following goodness-of-fit indicators recommended by Hu and Bentler [42]: a root mean square error of approximation (RMSEA) of ≤0.06; a standardized root mean square residual (SRMR) of ≤0.08; and a comparative fit index (CFI) and Tucker–Lewis index (TLI) of ≥0.95. Such criteria must adhere to guidelines, and values close to standards can be accepted, for example a CFI–TLI > 0.90 or an RMSEA of up to 0.10 [43].

Raykov’s reliability coefficient (RRC) was used [44,45,46]. RRC was good if it was ≥0.700 and acceptable if it was ≥0.600 [44,45,46]. In this study, RRC was computed using the Stata module developed by Mehmetoglu, namely the RELICOEF module [47].

According to Campbell and Fiske [48], convergent validity in a CFA is commonly referred to as the *Average Variance Extracted* (AVE) [49]. The AVE was considered good if it was ≥0.500 but if it was just under 0.500, as Fornell and Larcker [49] preconized, it was considered that the RRC needed to be higher than 0.600. In our study, the AVE was computed using a Stata^®^ module developed by Mehmetoglu [50], namely the *CONDISC* module. Discriminant validity was assessed using the *CONDISC* module [50]. Squared correlations (SC) were among the latent variables that were computed. If AVE values were ≥SC values, there was no problem with discriminant validity [51].

### 3.5. Convergent Validity

To establish the convergent validity of the AAS scale, we looked at the extent to which scores on our AAS hetero-reporting scale correlated with students’ scores on a scale measuring children’s feelings about school based on self-system theory [18], the French enriched version of this scale being validated in a previous study [30]. We chose this self-report scale to measure children’s feelings at school to establish our convergent validity, because it is based on the self-system theory [8,15,52,53], which is a sub-theory of the SDT we used for the AAS scale but applied to the field of education. We therefore conducted an analysis of the correlations between the French FAS score of a sample of children and the AAS score obtained for each of these same children, this time by soliciting their teachers.

### 3.6. Test–Retest

The scale was filled in twice by the teachers (i.e., at t0 and t1). Test–retest reliability was determined using the intraclass correlation coefficient (ICC) with a 95% confidence interval (CI) between t0 and t1. A two-way random-effects model was used as it was determined to be the most appropriate [54]. The ICC value is considered excellent when is >0.75, good when it is between 0.60 and 0.74, moderate when it is between 0.40 and 0.59, and poor when it is below 0.40 [54]. Here, the ICC values between T0 and T1 were excellent for the global score (ICC = 0.89; 95% CI = 0.87–0.92, *p* < 0.001).

### 3.7. ANOVA

Although the prerequisite of the normality of the variables was not observed, an ANOVA was performed to assess interindividual differences according to gender, educational level and school environment. It is well established that ANOVA is a fairly robust test representing a valid option in the case of a violation of the normality assumption [55]. The pre-requisite of the homogeneity of variances was checked using Levene’s test. Bonferroni post hoc tests were used to complete the analysis of variables with more than two modalities and indicating a significant global difference.

## 4. Results

### 4.1. Results of Face Validity

The analysis of the responses collected from our 26 expert participants (i.e., 10 master’s and 16 PhD students in psychology, including 9 males, 15 females, and 1 non-gender-specific individual) reassured us a priori about the scale’s apparent validity. Regarding the questions dealing with syntax, vocabulary, and the correct length of statements (i.e., item i and iv; see 3.1.), there were 100% favorable responses to scale items. Responses were largely supportive regarding clarity (i.e., 96%; item ii; 3.1.) but also grammar (i.e., 92%; item iii, 3.1.). Participants’ responses confirmed the lack of response orientation (i.e., 80%; item vi, 3.1.), including no bias towards what is socially desirable (i.e., 77%; item vii, 3.1.). Finally, the responses collected signaled the absence of discomfort with the words used (i.e., 77%; item v, 3.1.) in the different AAS scale items.

### 4.2. Results of Exploratory Factor Analysis (EFA)

Table 2 shows the means, standard deviations and 95% confidence intervals observed for each of the 11 analyzed items of the AAS scale (first sample; N = 164). Most of the item scores ranged from 0 to 10 (items 1, 4, 5, 6, 7, 8, 9, and 10). Item 2 scores ranged from 1.1 to 10, item 3 scores ranged from 0.5 to 10 and item 11 scores ranged from 2.2 to 10.

The Shapiro–Wilk normality test showed a violation of the assumption of normality for the 11 analyzed items (*p* < 0.0001). The Doornik–Hansen test also indicated a violation of multivariate normality (χ^2^ (22) = 156.666, *p* < 0.0001). Therefore, an EFA using a principal axis method was appropriate. The KMO measure of sampling adequacy (KMO = 0.93) and the Bartlett’s test of sphericity (χ^2^ (55) = 1851.273, *p* < 0.0001) indicated that the scale was psychometrically adequate for EFA. Moreover, the Spearman’s correlation matrix indicated significant inter-items correlations (*p* < 0.0001) comprised from ρ = 0.44 to *ρ* = 0.86 (Table 3).

**Figure 1:**
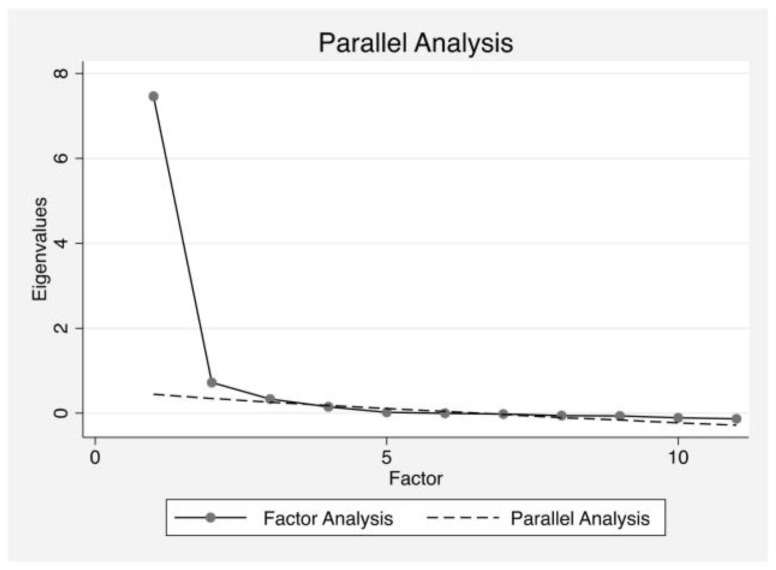
Scree plot and parallel analysis of eigenvalues for the AAS scale (11 items) factors.

Parallel analysis primarily suggested a unique factor scale with an eigenvalue higher than 1 (Table 4). However, the scree plot indicated two other factors with eigenvalues higher than those in the simulated data (Figure 1) but with a small difference between those from factor analysis and parallel analysis for the third factor (Δ = 0.072; Table 4). Based on these criteria, a forced one-factor EFA using PAF was first performed. Additionally, both forced two-factor and three-factor EFA using PAF with Promax rotation including Kaiser normalization (Kappa = 3) were performed.

For one-factor EFA all items loaded at least 0.657 on the unique factor (Table 5) explaining 72.98% of the variance and that could be named AAS, which showed α = 0.95.

2-factor EFA. Item 3 (“Interest for school work”) was removed due to high cross-loading (loading = 0.600 on factor 1 while loading = 0.387 on factor 2). After removing this item EFA-PAF with Promax rotation including Kaiser normalization (Kappa = 3) was repeated on the AAS-10 explaining 72.31% of the variance. KMO for this AAS-10 was excellent (KMO = 0.91) and the Bartlett test of sphericity was significant (χ^2^ (45) = 1575.337, *p* < 0.0001). All items load at least 0.640 on their respective factor (Table 5). Factor 1 comprising 6 items (1—Mathematics; 5—Pleasure to learn; 6—Graphic abilities; 7—Autonomy; 9—Literacy; 10—Perseverance) could be named “Performance & Autonomous Motivation (PAM)”. Factor 2 including 4 items (2—Adjustment with teacher; 4—Integrative skills; 8—Adjustment with peers; 11—Link with student) could be named “Social Adjustment (SA)”. In addition, all subscales yielded on reliable properties (Factor 1—PAM: α = 0.95; Factor 2—SA: α = 0.87). Finally, Factor 1- PAM was strongly correlated with Factor 2—SA after Promax rotation (*r* = 0.67).

3-factor EFA. Item 3 (“Interest for school work”) was removed due to high cross-loading (loading = 0.633 on factor 1 while loading = 0.339 on factor 2). After removing this item EFA-PAF with Promax rotation including Kaiser normalization (Kappa = 3) was repeated on the AAS-10 explaining 78.22% of the variance. KMO for this AAS-10 was excellent (KMO = 0.91) and Bartlett test of sphericity was significant (χ^2^ (45) = 1575.337, *p* < 0.0001). All items load at least 0.652 on their respective factor (Table 5). Factor 1 comprising 6 items (1—Mathematics; 5—Pleasure to learn; 6—Graphic abilities; 7—Autonomy; 9—Literacy; 10—Perseverance) could be named “Performance & Autonomous Motivation (PAM)”. Factor 2 including 2 items (4—Integrative skills; 8—Adjustment with peers) could be named “Student-Peer link (PP)”. Factor 3 including 2 items (2—Adjustment with teacher; 11—Link with student) could be named “Student Teacher link (PT)”. In addition, all subscales yielded on reliable properties (Factor 1—PAM: α = 0.95; Factor 2—PP: α = 0.90; Factor 3—PT: α = 0.83). Finally, all subscales were strongly intercorrelated after Promax rotation as follows: Factor 1—PAM with Factor 2—PP (r = 0.63); Factor 1—PAM with Factor 3—PT (*r* = 0.59); Factor 2—PP with Factor 3—PT (*r* = 0.61).

1-factor solution could be named “AAS”. 2-factor solution: Factor 1 could be named: “PAM”; Factor 2 could be named: “SA”. 3-factor solution: Factor 1 could be named: “PAM”; Factor 2 could be named: “PP”; Factor 3 could be named: “PT”.1-factor EFA. All items loaded at least 0.657 on unique factor (Table 5) explaining 72.98% of variance and that could be named AAS which showed α = 0.95.

### 4.3. Results of Confirmatory Factor Analysis (CFA)

#### 4.3.1. Multivariate Normality and Covariate Matrix

Table 2 shows means, standard deviations and 95% confidence intervals observed for each of the 11 analyzed items of AAS (second sample; N = 361). The Doornik-Hansen test indicated a violation of multivariate normality for both AAS-11 (χ^2^ (22) = 217.509, *p* < 0.0001) and AAS-10 (χ^2^ (20) = 206.370, *p* < 0.0001). CFA using Maximum Likelihood Robust method including a Satorra-Bentler correction was then performed to assess 1-factor model, 2-factor model and 3-factor model. Table 6 shows the covariance matrix between the 11 items.

#### 4.3.2. Goodness-of-Fit

**Figure 2:**
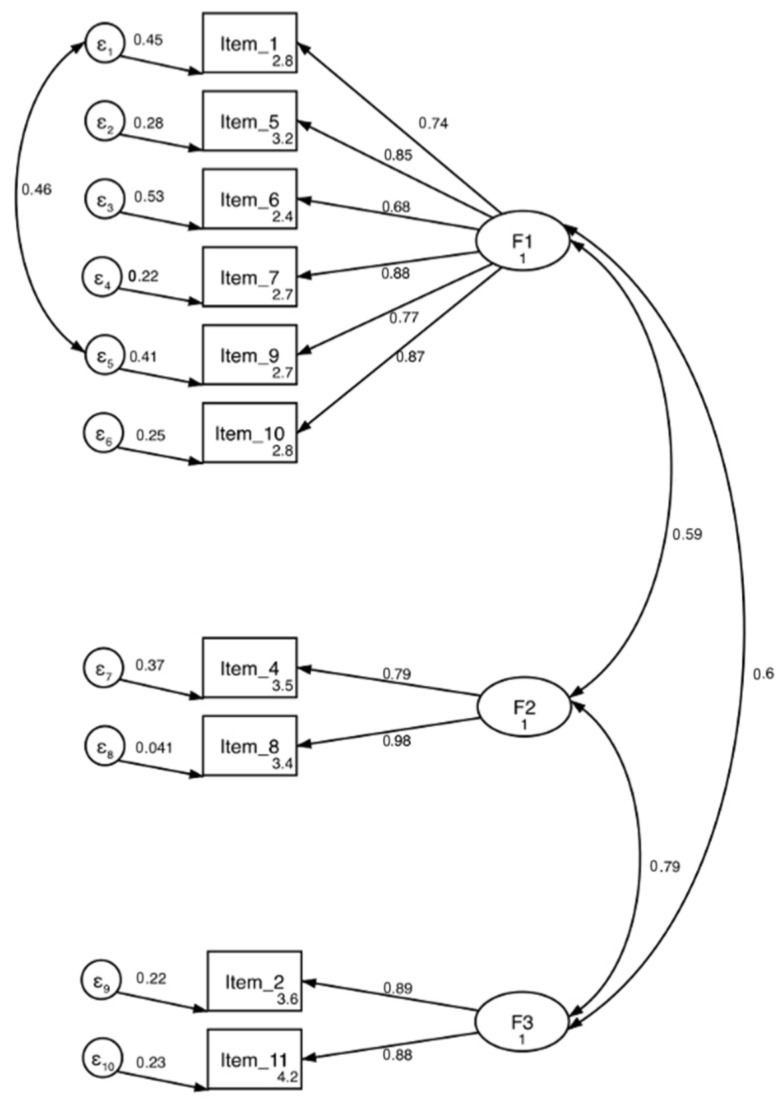
Structural model of the AAS with factors loadings and inter-factors correlates.

For all the models tested, an error covariance was added between “Mathematics” (item 1) and “Literacy” (item 9) because of the strong link that exists among young schoolchildren between success in mathematics and success in French. At this stage, academic success is more complete. The one-factor model and the two-factor model showed ab inadequate fit (Table 7). Conversely, the three-factor model was retained due to an adequate fit with the exception of a significant Satorra–Bentler Chi-square (χ^2^ SB = 81.181, df = 31, *p* < 0.001; RMSEA-SB = 0.067, CFI-SB = 0.974, TLI-SB = 0.963, SRMR = 0.049). Figure 2 shows the final model and all items’ significant loading on their expected factors.

#### 4.3.3. Reliability

For the retained three-factor model, all subscales showed good Raykov’s factor reliability coefficients (Factor 1—RRC = 0.899; Factor 2—RRC = 0.886; Factor 3—RRC = 0.874). AAS also showed good convergent validity (Factor 1—AVE = 0.644; Factor 2—AVE = 0.793; Factor 3—AVE = 0.775). All of these AVEs were also higher than the SC among latent variables (Factor 1/Factor 2—SC = 0.345; Factor 1/Factor 3—SC = 0.366; Factor 2/Factor 3—SC = 0.775), which showed good discriminant validity. The final model is reported in Figure 2.

### 4.4. Results of Convergent Validity

The sample on which these measures (i.e., scores from the French FAS and the AAS scales) were taken consisted of N = 283 children enrolled in elementary school (143 boys and 140 girls; 62 CP (i.e., French first grade), 71 CE1 (i.e., French second grade), 53 CE2 (i.e., French third grade), 47 CM1 (i.e., French fourth grade) and 50 CM2 (i.e., French fifth grade)). This allowed us to observe a positive correlation (*r* = 0.17; *p* < 0.01) between the two scales, which, given their similar theoretical anchoring, testifies to the convergent validity of the AAS scale for measuring children’s Autodetermination At School.

### 4.5. Interindividual Differences

Among the 361 evaluated children (sample 2), we obtained descriptive data for 344 of them (mean age = 7.64; SD = 1.53; range = 5–11) and no significant association was observed between age and Factor 1, Factor 2, Factor 3 or total score (*p* > 0.05). Table 8 shows gender, level and school environment differences for AAS’s total score and subscales. ANOVAs highlighted that girls had a significantly better relationship with teachers than boys did (*F* (1, 342) = 5.37, *p* < 0.05). Furthermore, we observed significant differences in school environment for total score (*F* (2, 341) = 5.18, *p* < 0.01), factor 1-PAM (*F* (2, 341) = 4.52, *p* < 0.05), factor 2-PP (*F* (2, 341) = 3.05, *p* < 0.05) and factor 3-PT (*F* (2, 341) = 3.64, *p* < 0.05). The Bonferroni post hoc test showed that higher scores were observed for schools located in non socio-economically disadvantaged areas, in comparison with schools located in disadvantages aeras, and this was the case for the total score (*t* = −7.46, *p* < 0.01), Factor 1 (*t* = −4.994, *p* < 0.05) and Factor 3 (*t* = −1.43, *p* < 0.05). No significant difference was observed according to school level.

## 5. Discussion

### 5.1. Summary of Key Findings

To validate the AAS scale, the methodology involved conducting face validity analysis, an exploratory factor analysis (EFA; n = 164), confirmatory factor analysis (CFA; n = 361), convergent validity analysis, test–retest analysis, and interindividual analysis (see Table 9). Reduced from 11 to 10 items, the scale exhibits a three-dimensional structure, allowing for a global score of school engagement and three distinct sub-scores related to the child’s performance and intrinsic motivation (Factor 1—PIM) and social engagement with peers (Factor 2—SP) and with the teacher (Factor 3—ST). The results emphasize a significant link between intrinsic motivation and the child’s performance while underscoring the importance of separately considering social engagement with peers and with the teacher to understand the functioning of elementary school students. Additionally, interindividual differences reveal the impact of the school environment on the AAS, with lower scores in disadvantaged schools. Gender differences also affect the teacher–student relationship, where boys’ scores are lower than those of girls in Factor 3, which is related to social engagement with the teacher.

### 5.2. Factorial Structure of the Scale

As each AAS item was based on the robust SDT [8], validity of scale was expected. However, the factor structure obtained is slightly different from what was predicted. The distribution expected should distinguish the teacher’s perception of student’s autonomous motivation (i.e., as the first factor), academic results (i.e., competence as the second factor) and finally, the child’s social abilities (i.e., as the third factor). The extraction of the factors into their main components revealed another interesting factor structure. Note that the factor structure of the AAS scale is ternary, as originally intended. According to a statistical study conducted, the AAS scale includes a first factor (F1-PAM) on child academic engagement. This dimension regroups performance and autonomous motivation together, both with items relating to intrinsic and internalized extrinsic motivations (items 5, 7 and 10) or IM [26] and to academic performance (items 1, 6 and 9). Secondly, the AAS results consider child social adjustment divided into two distinct factors (F2-PP and F3-PT). F2, via items 4 and 8, corresponds to a schoolchild’s commitment in relationship with peers. F3, via items 2 and 11, refers independently, to the teacher’s perception of student commitment in relationship with the teacher (i.e., with the questionnaire’s respondent). Hence, this result of component extraction is interesting in two respects. On the one hand, it reaffirms how much intrinsic motivation and academic performance are closely linked in terms of commitment [7,56]. It also reaffirms how much the satisfaction of basic psychological needs such as autonomy [6,34,38] and competence is directly correlated to intrinsic motivation [8,55] and especially the perceptible measurement of pleasure or tenacity [26] for learning. However, it is very important to note that this close bond has been observed here in the French educational system, which is still very hierarchical, where academic success, easily assessable by the child through evaluative feedback, can largely explain the maintenance of the student’s appetite for learning, with achieving good evaluations holding the value of an objective. On the other hand, it underlies two aspects to be distinguished in order to understand a schoolchild’s social commitment at school, which is not a uniform whole. Teachers’ and peers’ relationships represent essential but separate dimensions considering the classroom’s social ecology [1]. Considering the lack of studies devoted to exploring the role of commitment with peers in global engagement at school [57], it is interesting to observe this phenomenon, which encourages us to split these two facets of social school engagement in this scale. This could also allow for the examination of socially divided commitment in certain populations with atypical development. For instance, studying it in gifted children could make sense, as their academic engagement appears to be linked to the quality of the relationship they have with their teacher rather than the one they have with their peers [58].

### 5.3. External Validity

An interindividual analysis of the AAS score and sub-scores suggests an impact of some independent variables on a young student’s engagement at school.

This starts with gender, because according to teachers, the student–teacher link is significantly better with girls than boys. This result is in line with the larger success in school for young girls, which is largely reported in the literature [59]. Looking for the origin of these gender differences naturally means questioning the influence of stereotypes and socialization values at school [60]. At school, girls (from age 4) and boys (from age 7) think adults consider boys academically inferior to girls and vice versa [61].

The school’s socioeconomic area also influences the AAS score. Some differences emerge between the scores of children who attend schools in middle socioeconomic areas and children who attend schools in low socioeconomic areas. The more the school is in a disadvantaged area, the more the overall AAS score, as well as the sub-scores obtained from the F1-PAM (i.e., about performance and autonomous motivation) and the F2-PT (i.e., about the teacher–student relationship) fall.

Finally, there is a trend evolution between the AAS score and the child level: the child’s commitment increases slowly each year from first grade to third and fourth grade, before falling back in fifth grade. This trend has been confirmed in another study we conducted with this scale, for the sub-dimension F2-PT (i.e., associated with the teacher–student relationship) [3].

### 5.4. Limitations and Research Perspectives

A limitation is that teachers report observed engagement instead of asking children directly about their perceived engagement. Even the teachers are in a good position to evaluate children’s commitment [13] based on their daily observation in the school context, and it could be relevant to confront their perception to those of children who can testify about their perception of engagement. The relevance of asking children directly about their experiences of commitment in school to examine whether teacher observation is correlated with children’s perceptions of their commitment in school has been already investigated in a recent study. In this study, using the French FAS [30] and the AAS, an interesting gap between gifted students and teacher perception of their relationship has been revealed that has not been observed for non-gifted students [3]. Incidentally, this result confirms the interest of the existence of this scale to study inter-individual differences in commitment at school, in the field of differential psychology.

The interindividual analyses conducted also call for the replication of these results and further examination, in subsequent research, of the explanatory factors for these differences in school commitment based on gender, socioeconomic factors, and the considered academic level. Indeed, it would be necessary to determine whether there is an effect of stereotypes on the perception of students’ commitment by their teachers, or if there is indeed a differential in commitment that needs to be addressed.

### 5.5. Applicability Perspectives

The AAS hetero-evaluative scale was designed to measure through the teacher’s prism the commitment in learning for children aged 6 to 12, thus raising awareness to consider the importance of commitment concepts in learning. This scale is an ecological and multidimensional tool with which to assess Autodetermination At School (AAS), both socially and academically. The AAS scale is available not only for researchers in psychology and education, but also for those in the field, to be used in an applied way (i.e., by educational, behavioral and clinical psychologists or child psychiatrists). Indeed, this scale may be especially useful to work with children who are perceived to be struggling at school. This scale could be a solution to the challenges faced by school psychologists, such as the lack of time and resources, as highlighted by Buttard [62]. It would enable an easy assessment of the student’s level of engagement in school, as well as the potential effectiveness of measures implemented to promote this commitment. Moreover, this scale has been designed to allow for test–retest reliability. It involves the child’s teacher, who has to respond to questions about his or her student. It is a way to encourage collaborations between teachers and psychologists; as follows, various professionals of childhood education can all be vigilant towards and support the harmonious development of the child. AAS is, therefore, an engaging scale for measuring schoolchild commitment. It encourages all the adults involved to go in the same direction and encourages concrete evaluation.

Furthermore, the results obtained in the interindividual analysis during the external validation of the scale highlighted the need to address the effects of gender and social background on the academic commitment of young learners. It is crucial to communicate these findings to education professionals, including education inspectors, school headmasters, and teachers, to make them aware of the presence of these effects. These effects may be the result of stereotyping, or may indicate a real decline in academic commitment among boys, younger pupils, and those enrolled in schools located in socio-economically disadvantaged areas, emphasizing the need for attention and intervention. This latter result also highlights the inadequacy of education policies implemented to remedy the detrimental effects of territorial inequalities on access to education. In this regard, one could envisage the creation, implementation, and evaluation of specific programs (using the AAS scale, for example) to test their effects on supporting the maintenance of learners’ commitment in these sensitive areas.

## 6. Conclusions

Without learning effects, the AAS scale allows repeated use for intra-individual, inter-individual or inter-group measures. Its factor structure was validated with an EFA and a CFA before conducting interindividual analysis to establish its external validity. Dimensions of the AAS are already evocative. It helps to understand a child’s autodetermination according to an academic and motivational factor (F1-PAM), but also thanks to a nuanced apprehension of a student’s social adjustment, through two distinct factors. Factor two (F2-PP) is about a child’s ability to link with peers. Factor three (F3-PT) is about their ability to show appropriate behavior to have a good relationship with their teacher. Moreover, interindividual analyses provide interesting results about the effects or trends of effects of gender, grade and school environment on student school engagement.

Evermore, the AAS scale is an interesting realistic and ecological tool, designed to have real applicability [32]. It is a promising scale with which to draw links between fields of psychology and education, between the psychologist and the teacher. It is a scale evaluating commitment in a coherent perspective to support childhood actors to overcome academic or social difficulties encountered at school. Its use and rating are facilitated since each item generates a significant score out of 10. These rates can then be added together to give an overall AAS score out of 100. Autodetermination At School can then be perceived as a useful tool with which to assess children’s commitment at school.

To conclude, the AAS scale facilitates unique or repeated measurement and the subsequent monitoring of the intra-personal progression of a child’s academic and social engagement in a school environment. Consequently, this hetero-evaluative scale seems as useful for researchers as it is for educational psychologists, behaviorists and/or clinicians in charge of children. Researchers can use it to explore some effects on school commitment of experimental programs, and new academic and/or pedagogical methods. They can still use this scale in differential psychology to uncover differences in engagement in the school environment between children with typical and atypical or troubled development. Overall, to support a schoolchild in difficulty, it could be helpful both for teachers and psychologists to monitor how the child’s commitment in school evolves, as a function of the educational program developed to respond to their individual needs.

## Figures and Tables

**Table 1:** Presentation of the 11 initial items.

Item	Question	Cursor at 0	Cursor at 10
**1—Mathematics**	How would you rate his/her level of achievement in mathematics? (Comment évaluez-vous son niveau de réussite en mathématiques?)	Far below (Très en dessous)	Far above (Très au-dessus)
**2—Adjustment to teacher**	How would you rate his/her attitude towards you? (Comment évaluez-vous son attitude à votre égard?)	Very poorly adjusted (Très mal ajustée)	Very well adjusted (Très bien ajustée)
**3—Interest**	How would you rate his/her interest in school work? (Comment évaluez-vous son intérêt pour le travail scolaire?)	Non-existent (Inexistant)	Very strong (Très fort)
**4—Integrative** **skills**	How would you rate his/her ability to integrate himself into the class group? (Comment évaluez-vous sa capacité d’intégration au groupe classe?)	Very bad (Très mauvaise)	Very good (Très bonne)
**5—Pleasure** **to learn**	How do you think he/she feels when you put him/her in a learning situation? (Que pensez-vous qu’il/elle éprouve lorsque vous le/la placez en situation d’apprentissage?)	A lot of displeasure (Beaucoup de déplaisir)	A lot of fun (Beaucoup de plaisir)
**6—Graphical** **ability**	How do you evaluate his/her writing from a graphic point of view? (Comment évaluez-vous son écriture d’un point de vue graphique?)	Very laborious (Très laborieuse)	Very neat (Très soignée)
**7—Autonomy**	Would you say that this student can work independently? (Diriez-vous de cet/cette élève qu’il/elle arrive à travailler en autonomie?)	Never (Jamais)	All the time (Tout le temps)
**8—Adjustment** **with peers**	How would you rate his/her attitude towards other children in the class? (Comment évaluez-vous son attitude à l’égard des autres élèves de sa classe?)	Very poorly adjusted (Très mal ajustée)	Very well adjusted (Très bien ajustée)
**9—Literacy**	How would you rate his/her level of success in literature? (Comment évaluez-vous son niveau de réussite en français?)	Far below (Très en dessous)	Far above (Très au-dessus)
**10—Perseverance**	Is this student discouraged by work? (Cet/cette élève se décourage-t-il/elle face au travail?)	All the time (Tout le temps)	Never (Jamais)
**11—Teacher–student**	How would you rate your relationship with the student? (Comment évaluez-vous le lien que vous entretenez avec cet/cette élève?)	Very bad (Très mauvaise)	Very good (Très bonne)

**Table 2:** Descriptive analysis for AAS.

Item	Sample 1 (N = 164)		Sample 2 (N = 361)
	Mean (*SD*)	95% CI	Mean (*SD*)
1—Mathematics	5.72 (3.25)	5.22–6.22	6.71 (0.13)
2—Adjustment with teacher	7.63 (2.23)	7.29–7.98	7.68 (0.11)
3—Interest for school work	6.35 (2.99)	5.89–6.81	7.24 (0.12)
4—Integrative skills	6.84 (2.60)	6.44–7.24	7.38 (0.11)
5—Pleasure to learn	6.13 (2.76)	5.70–6.55	7.09 (0.12)
6—Graphic abilities	5.92 (3.22)	5.43–6.42	6.68 (0.14)
7—Autonomy	5.80 (3.59)	5.25–6.35	7.05 (0.14)
8—Adjustment with peers	6.91 (2.50)	6.52–7.29	7.36 (0.11)
9—Literacy	5.67 (3.36)	5.15–6.19	6.80 (0.13)
10—Perseverance	5.83 (3.33)	5.32–6.34	6.94 (0.13)
11—Link with student	7.78 (2.05)	7.47–8.10	7.95 (0.10)

**Table 3:** Spearman’s ranks correlations for AAS (N = 164).

Item	1	2	3	4	5	6	7	8	9	10	11
**1**	-										
**2**	0.44 ***	-									
**3**	0.75 ***	0.65 ***	-								
**4**	0.61 ***	0.51 ***	0.64 ***	-							
**5**	0.79 ***	0.51 ***	0.86 ***	0.61 ***	-						
**6**	0.61 ***	0.45 ***	0.64 ***	0.54 ***	0.63 ***	-					
**7**	0.81 ***	0.51 ***	0.78 ***	0.63 ***	0.81 ***	0.74 ***	-				
**8**	0.58 ***	0.57 ***	0.66 ***	0.77 ***	0.62 ***	0.54 ***	0.66 ***	-			
**9**	0.84 ***	0.48 ***	0.78 ***	0.60 ***	0.82 ***	0.73 ***	0.86 ***	0.64 ***	-		
**10**	0.76 ***	0.56 ***	0.78 ***	0.58 ***	0.84 ***	0.63 ***	0.80 ***	0.60 ***	0.79 ***	-	
**11**	0.49 ***	0.70 ***	0.65 ***	0.57 ***	0.57 ***	0.49 ***	0.53 ***	0.67 ***	0.55 ***	0.55 ***	-

Notes. ***: *p* < 0.0001. 1: mathematics; 2: adjustment to teacher; 3: interest for school work; 4: integrative skills; 5: pleasure to learn; 6: graphic abilities; 7: autonomy; 8: adjustment to peers; 9: literacy; 10: perseverance; 11: link with student.

**Table 4:** Parallel analysis for factor analysis (N = 164).

Factors	Factor Analysis	Parallel Analysis	Difference
**1**	**7.462**	**0.447**	**7.016**
**2**	**0.722**	**0.347**	**0.375**
**3**	**0.332**	**0.261**	**0.072**
4	0.151	0.182	−0.031
5	0.024	0.110	−0.087
6	−0.001	0.042	−0.044
7	−0.020	−0.029	0.010
8	−0.056	−0.104	0.048
9	−0.064	−0.158	0.094
10	−0.108	−0.227	0.119
11	−0.131	−0.281	0.150

**Table 5:** Factor loadings for the 1-factor solution, the 2-factor solution and the 3-factor solution of AAS determined by performing EFA (N = 164).

Items	1-Factor	2-Factor ^1^		3-Factor ^1^		
		Factor 1	Factor 2	Factor 1	Factor 2	Factor 3
**1—Mathematics**	**0.845**	**0.844**	0.047	**0.8435**	0.0651	−0.016
**2—Adjustment with teacher**	**0.657**	0.055	**0.706**	0.090	0.101	**0.654**
**3—Interest for school work**	**0.913**	-	-	-	-	-
**4—Integrative skills**	**0.721**	0.191	**0.640**	0.143	**0.757**	0.006
**5—Pleasure to learn**	**0.889**	**0.796**	0.136	**0.807**	0.027	0.110
**6—Graphic abilities**	**0.739**	**0.642**	0.147	**0.652**	0.064	0.085
**7—Autonomy**	**0.913**	**0.883**	0.085	**0.885**	0.119	−0.033
**8—Adjustment with peers**	**0.760**	0.101	**0.799**	0.016	**0.876**	0.114
**9—Literacy**	**0.905**	**0.948**	0.007	**0.944**	0.032	−0.023
**10—Perseverance**	**0.865**	**0.790**	0.125	**0.814**	−0.039	0.153
**11—Link with student**	**0.693**	0.075	**0.732**	0.040	0.022	**0.881**
**% of explained variance**	72.98	65.35	6.96	65.84	8.00	4.38
**Cronbach’s α**	0.95	0.95	0.87	0.95	0.90	0.83

Notes. Factor loadings above 0.30 appear in bold. Extraction: principal axis factoring. ^1^ Rotation Promax with Kaiser normalization (Kappa = 3).

**Table 6:** Variance—Covariance matrix (N = 361).

Item	1	2	3	4	5	6	7	8	9	10	11
**1**	**5.761**										
**2**	1.602	**4.491**									
**3**	3.481	3.163	**5.611**								
**4**	1.548	2.353	2.504	**4.430**							
**5**	3.606	2.441	4.482	2.276	**4.989**						
**6**	3.017	2.417	3.709	1.995	3.440	**7.472**					
**7**	4.284	2.585	4.431	2.673	4.296	4.408	**6.871**				
**8**	1.357	3.125	2.795	3.523	2.428	2.670	2.992	**4.654**			
**9**	4.693	1.886	3.786	1.698	3.883	3.870	4.515	1.861	**6.521**		
**10**	3.796	2.811	4.372	2.508	4.026	3.942	4.988	2.710	4.143	**6.083**	
**11**	1.196	3.143	2.504	2.167	2.117	1.836	2.156	2.855	1.455	2.146	**3.661**

Notes. Variance results appear in bold. 1: mathematics; 2: adjustment with teacher; 3: interest for school work; 4: integrative skills; 5: pleasure to learn; 6: graphic abilities; 7: autonomy; 8: adjustment with peers; 9: literacy; 10: perseverance; 11: link with student.

**Table 7:** Fit indices of multiple CFA of the AAS (N = 361).

	χ*^2^*_SB_ (*df*)	*p*	χ*^2^*_SB_/*df*	RMSEA_SB_	CFI_SB_	TLI_SB_	SRMR	AIC	BIC
**1-factor model**	502.152 (43)	<0.0001	11.678	0.172	0.802	0.747	0.105	15,465.177	15,597.399
**2-factor model**	157.011 (33)	<0.0001	4.758	0.102	0.936	0.913	0.055	13,870.062	13,994.506
**3-factor model**	81.181 (31)	<0.001	2.619	0.067	0.974	0.963	0.049	13,763.885	13,896.106

Notes. AIC = Akaike information criterion; BIC = Bayesian information criterion; CFI_SB_ = Satorra–Bentler comparative fit index; RMSEA_SB_ = Satorra–Bentler root mean square error of approximation; SRMR = standardized root mean square residual; TLI_SB_ = Satorra–Bentler Tucker–Lewis Index. All tested models comprise an error covariance between item 1 (mathematics) and item 9 (literacy).

**Table 8:** Interindividual differences.

		Total Score AAS	Factor 1—PAM	Factor 2—PP	Factor 3—PT
		F (df1, df2)/Mean (*SD*)	F (df1, df2)/Mean (*SD*)	F (df1, df2)/Mean (*SD*)	F (df1, df2)/Mean (*SD*)
**Total sample**		71.45 (17.73)	41.19 (12.67)	14.72 (4.02)	15.54 (3.82)
**Gender**		**2.00 (1, 342)**	**0.34 (1, 342)**	4.85 (1, 342) *	5.37 (1, 342) *
Boys (n = 174, 50.58%)		70.11 (18.03)	40.79 (12.55)	14.25 (4.27)	15.07 (4.03)
Girls (n = 170, 49.42%)		72.81 (17.37)	41.59 (12.82)	15.20 (3.71)	16.02 (3.54)
**Level**		**1.64 (4, 339)**	**1.22 (4, 339)**	**1.26 (4, 339)**	**2.30 (4, 339) ^†^**
1st grade (n = 81, 23.55%)		69.94 (18.04)	40.72 (12.93)	14.35 (4.22)	14.87 (3.97)
2nd grade (n = 82, 23.84%)		68.89 (17.88)	39.75 (13.19)	14.11 (3.91)	15.03 (4.03)
3rd grade (n = 56, 16.28%)		76.25 (17.44)	44.42 (11.66)	15.39 (4.51)	16.44 (3.64)
4th grade (n = 63, 18.31%)		72.14 (17.22)	40.82 (12.55)	15.12 (3.72)	16.21 (3.37)
5th grade (n = 62, 18.02%)		71.75 (17.54)	41.15 (12.52)	15.00 (3.70)	15.61 (3.75)
**School environment**		5.18 (2, 341) **	4.52 (2, 341) *	3.05 (2, 341) *	3.64 (2, 341) *
No classification (n = 215, 62.50%)		73.68 (17.40)	42.68 (12.07)	15.13 (4.01)	15.87 (3.80)
Disadvantaged (n = 62, 18.02%)		69.34 (17.08)	39.79 (13.48)	13.97 (3.80)	15.58 (3.51)
Very disadvantaged (n = 67, 19.48%)		66.22 (18.30)	37.69 (13.12)	14.09 (4.15)	14.44 (3.99)

Notes. ^†^: *p* < 0.10; *: *p* < 0.05; **: *p* < 0.01.

**Table 9:** Constellation of results for final AAS.

Analysis Performed	Recommendations	Results for AAS	Valid or Not?
**Face validity**	>70% of agreement per item	100% for items i and iv; 96% for ii; 92% for iii; 80% for vi; 77% for v et vii	Valid
**EFA**	Validation of a 3-factorconstruct for a scale of 11 items	Removal of item 3. The AAS-10 explains 78.22% of the variance A 3-factor construct obtained: Factor 1: “PAM”; Factor 2: “PP”; Factor 3: “PT”.	Valid and interesting but different in the expected construct. A 10-item scale.
**CFA**	Confirmation of the factor structure obtained with the EFA and assessment of construct validity	Goodness-of-fit for the 3-factor model Reliability: Good discriminant validity for the 3-factor model	Valid
**Convergent validity**	To observe a correlation with the French FAS scale.	There is a link between the French FAS and the AAS (r = 0.17; *p* < 0.01)	Valid
**ANOVA**	To observe interindividual differences	Gender, level and school environment differences	Valid

## Data Availability

Data is contained within the article.
